# A scenario of left ventricular assist device suction mimicking external compression

**DOI:** 10.1007/s12055-025-02000-y

**Published:** 2025-07-18

**Authors:** Branislav Bezak, Panagiotis Artemiou, Matej Ondrusek, Stefan Durdik, Ivo Gasparovic, Michal Hulman

**Affiliations:** 1 Faculty of Medicine, Department of Cardiac Surgery, Comenius University, National Institute of Cardiovascular Diseases, Pod Krasnou Horkou 1, Bratislava, 83101 Slovakia; 2 Faculty of Medicine, Department of Surgical Oncology, Comenius University, St. Elizabeth Oncology Institute, Bratislava, Slovakia

**Keywords:** HeartMate 3, Pericardial tamponade, Left ventricular assist device, Suction event, Pump speed, Pump power

## Abstract

In this short report, we present a rare scenario involving a 60-year-old male patient, in whom higher pump speed and suction mimicked external compression of the left ventricle. Surgical revision revealed excessive suctioning of the left ventricle and left atrium by the left ventricular assist device, leading to atrioventricular groove distortion that mimicked external compression of the left ventricle. After adjustment of the pump speed, obstruction of the inflow cannula and collapse of the left ventricle were resolved.

## Background

Common etiologies for reduced left ventricular assist device (LVAD) flows include bleeding, dehydration, pericardial tamponade, right ventricular dysfunction, inflow or outflow occlusion, and suction events. During suction events, the ipsilateral ventricular cavity collapses around the ventricular assist device cannula [1–3]. The most common causes of suction include volume depletion, tamponade, and inflow cannula malposition. Pulsatility markers may be either low or extremely high in combination with low-flow alarms. Power is also directly related to device speed and flow, and suction events typically lead to a drop in both ventricular assist device flow and pump power [4].

In this short report, we present a rare scenario where higher pump speed and suction mimic external compression of the left ventricle.

## Case report

A 60-year-old patient with end-stage ischemic cardiomyopathy (New York Heart Association-class IV, INTERMACS profile 3) underwent implantation of a left ventricular assist device (LVAD; HeartMate 3, HM 3, Abbott Laboratories, Abbott Park, IL) in December 2024, due to exhausted conservative treatment options and left atrial appendage occlusion (AtriClip Flex 50 mm, AtriCure Inc, Amsterdam, Netherlands) with full sternotomy.

The patient left the operating room in stable hemodynamic condition with the following parameters: mean arterial pressure (MAP) 75 mmHg, pump speed 5200 rounds per minute (RPM), pump power 3.9 W, pump flow 4.5 L/min, and pulsatility index 2.7.

In the early postoperative period (under echocardiographic guidance), in an effort to improve end-organ perfusion, LVAD speed was gradually increased to 5800 RPM. However, this was accompanied by a progressive decrease in pump flow to 3.5 L/min. Additionally, MAP, pump power, and power index (PI) decreased to 50 mmHg, 3.6 W, and 1.2, respectively. The patient’s fluid balance was negative, at –290 mL. The International normalization ratio and serum creatinine levels were 1.13 and 162 µmol/L, respectively.

**Fig. 1:**
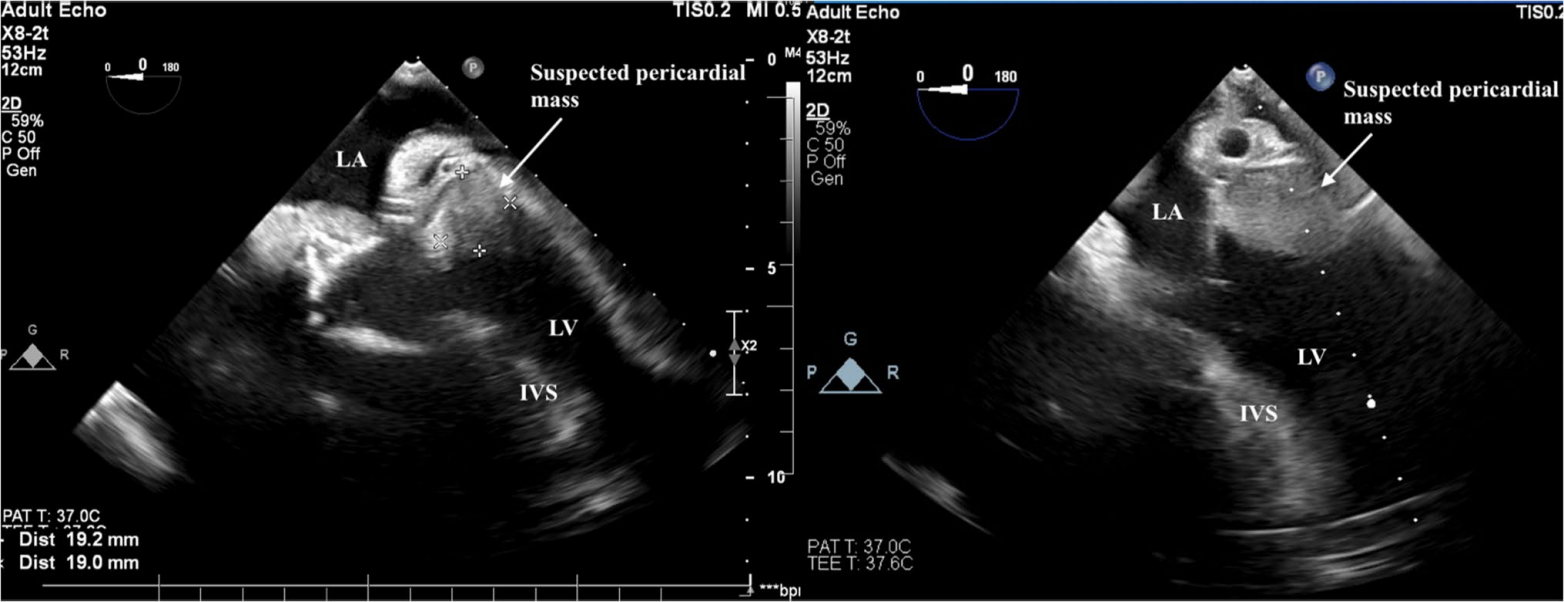
Echocardiography showing external compression to the left ventricle. LA, left atrium; LV, left ventricle; IVS, interventricular septum

Echocardiography performed in the intensive care unit revealed right ventricular failure and left ventricular compression, likely due to a pericardial mass (Fig. 1).

The patient was taken back to the operating room for emergency surgical revision due to suspected external compression. The sternum was reopened, and intraoperative findings revealed excessive suction of the subannular portion of the left ventricle and left atrium by the LVAD, leading to atrioventricular groove distortion that mimicked external left ventricular compression.

Cardiopulmonary bypass (CPB) was established via peripheral cannulation to enable rapid initiation of CPB, as the patient was in a state of cardiogenic shock. Additionally, the presence of the LVAD outflow cannula in the ascending aorta could have impeded aortic cannulation.

After reperfusion, fluid status optimization, and balanced inotropic support with dobutamine, adrenaline, and noradrenaline, the patient was weaned from CPB and returned to the intensive care unit. The LVAD parameters were as follows: MAP 80 mmHg, central venous pressure 12 mmHg, pulmonary capillary wedge pressure 14 mmHg, systemic vascular resistance 1350 dyn/s/cm^5^, pump speed 5000 RPM, pump power 3.3 W, pump flow 4.2 L/min, and pulsatility index 2.8. Optimization of volume status, pump speed, and inotropic support helps restore preload reserve, support left ventricular filling, and optimize LVAD function.

Following the revision, the patient recovered well and was extubated after 3 days.

Informed consent was obtained from the patient for publication of this case report.

## Discussion

This case describes a rare complication following LVAD implantation, characterized by excessive suctioning of the left ventricle and left atrium, leading to atrioventricular groove distortion and mimicking pericardial tamponade or external compression.

Excessive suction events in LVAD patients can present clinically as external cardiac compression or tamponade. These events occur when the LVAD’s drainage exceeds venous return, causing collapse of the left ventricular walls and potential obstruction of the inflow cannula. This can result in decreased cardiac output and hemodynamic instability [1–3].

The literature contains limited data regarding this rare complication. Moscato et al. [1] highlight that ventricular suction events can damage the myocardial wall, induce ventricular arrhythmias, and cause thromboembolic events due to disturbed blood flow during abnormal pump operation.

Echocardiographic assessments are crucial in identifying suction events. Indicators include excessive left ventricular decompression, leftward shift of the interventricular septum, and a reduced left ventricular cavity size — findings that can mimic the echocardiographic signs of cardiac tamponade [5]. Prompt recognition and management are critical, as delayed intervention could result in significant morbidity or mortality. Surgeons and intensivists should maintain a high index of suspicion for such scenarios in patients presenting with hemodynamic instability after LVAD implantation. Differentiating between suction events and external compressive forces is very important [6].

Management strategies for suction events involve adjusting the LVAD pump speed to ensure adequate left ventricular filling and prevent collapse. In some cases, temporarily reducing or stopping the pump can rapidly reverse the collapse. Additionally, optimizing volume status and right ventricular function is essential to prevent recurrent suction events [7].

Moreover, in the future, suction modules can be used to investigate suction in different pathophysiological conditions and to support the development of LVAD physiological controllers [8].

## Conclusions

Despite vigilant postoperative care and careful echocardiographic assessment, awareness of this rare complication can aid in distinguishing it from tamponade or external compression, ensuring timely and appropriate management to optimize patient outcomes.

## Data Availability

The data supporting the findings of this study are available within the article. Additional data are available from the corresponding author upon reasonable request.
